# Supplementary material for: Pre-Partum Supplementation with Polyunsaturated Fatty Acids on Colostrum Characteristics and Lamb Immunity and Behavior after a Mild Post-Weaning Aversive Handling Period
Source: Animals (Basel). 2022 Jul 11;12(14):1780. doi: 10.3390/ani12141780 (PMC9311828; doi:10.3390/ani12141780)
Supplement: Supplementary file 1 [file animals-12-01780-s001.zip › animals-1735729-supplementary.pdf]

Table S1. Ingredients and chemical composition of experimental diets; concentrates, hay sainfoin and fescue hay.

	CO-FES[1]		ALA-SAIN[2]		CO-FES/ ALA-SAIN
**Item (% of dry matter)**	**Concentrate CO**	**Hay FES**	**Concentrate ALA**	**Hay SAIN**	**Silage CORN**
**Ingredients**					
DDGs Corn (10% FA[3])	29.91	-	0	-	-
Oats	25.0	-	18.0	-	-
Linseed meal (28% FA[3])	0	-	20.0	-	-
Corn	15.0	-	0	-	-
Soybean meal	12.0	-	15.0	-	-
Barley	10.4	-	37.3	-	-
CPRC	0	-	5	-	-
Molasses	3.0	-	3.0	-	-
Hydrogenated palm fat By-Pass	3.0	-	0	-	-
Sodium bicarbonate	1.0	-	1.0	-	-
Calcium carbonate	0.19	-	0.17	-	-
Dicalcium phosphate	0.10	-	0.10	-	-
Vitamin-mineral premix[4]	0.4	-	0.4	-	-
**Chemical composition**					
Dry matter	90.1	84.9	89.8	84.9	43.9
Organic matter	94.9	89.9	95.5	94.8	94.9
Crude protein	20.2	9.9	16.8	15.7	6.0
Neutral detergent fibre	-	54.3	-	42.7	38.0
Acid detergent fibre	-	28.2	-	32.5	20.0
Fat	8.3	1.3	9.0	1.2	-
Crude fibre	6.3	25.9	5.8	31.7	-
Starch	26.9	-	31.8	-	25.1
FUL[5]	1.13		1.19		
**Fatty acids, g/100g FA[3]**					
12:0	0.21		0.41		
14:0	0.48		0.42		
15:0	0.04		0.09		
16:0	41.89		14.17		
17:0	0.11		0.16		
18:0	1.64		4.20		
20:0	0.42		0.50		
21:0	0.01		0.04		
22:0	0.17		0.40		
24:0	0.19		0.29		
cis-9 16:1	0.11		0.23		
cis-9 18:1	20.85		29.74		
cis-11 18:1	0.71		2.23		
cis-11 20:1	0.34		0.72		
18:2*n*-6	30.64		22.17		
18:3*n*-3	1.70		23.79		
cis-15 24:1	0.44		0.38		

[1]CO: Control concentrate; FES: Fescue hay (*Festuca pratensis*).

[2]ALA: Alpha-Linolenic Acid concentrate; SAIN: Sainfoin hay (*Onobrychis viciifolia*).

[3]FA: fatty acids.

[4]Vitamin and mineral premix contained per kg of DM: 2500 IU of vitamin A, 400 mg of vitamin D, 2.5 IU of vitamin E, 4.9 mg of Zn, 4.05 mg of Mn and 0.1 mg of Se (Calseaphos, Saint Malo, France).
[5]FUL: Feed unit for lactation.

Table S2. Effect of pre-natal diet on colostrum saturated fatty acid composition.

	Pre-natal diet				
	CO-FES[1]		ALA-SAIN[2]		
**Variable**	**Mean**	**SE**	**Mean**	**SE**	**P-value**
**Saturated fatty acids (SFA; mg/g total fat)**					
4:0	32.80	2.04	34.83	2.10	0.535
6:0[3]	8.62	0.75	10.22	0.66	0.157
8:0	4.92	0.64	6.67	0.66	0.099
10:0	12.63	2.26	18.91	2.36	0.093
11:0	0.10	0.03	0.15	0.03	0.232
12:0[3]	12.92	0.78	16.04	0.93	0.050
12:0 *iso*[3]	0.11	0.02	0.14	0.03	0.762
12:0 *anteiso*	0.10	0.01	0.09	0.01	0.693
13:0[3]	0.27	0.02	0.37	0.05	0.654
13:0 iso[3]	0.46	0.05	0.41	0.05	0.323
14:0	63.32	6.45	84.00	6.49	0.065
14:0 *iso*	1.28	0.12	1.11	0.12	0.364
14:0 *anteiso*	1.79	0.25	1.78	0.25	0.962
15:0	3.20	0.48	4.93	0.50	0.035
15:0 *iso*	1.45	0.13	1.26	0.13	0.390
16:0[3]	216.3	11.09	231.3	13.02	0.315
16:0 *iso*[3]	0.11	0.02	0.09	0.02	0.672
16:0 *anteiso*	2.89	0.14	3.22	0.14	0.167
17:0	4.92	0.33	5.68	0.32	0.163
18:0	71.99	5.79	65.54	5.74	0.491
20:0[3]	2.17	0.12	1.94	0.15	0.306
21:0	0.44	0.05	0.59	0.05	0.092
22:0	0.38	0.06	0.50	0.06	0.227
23:0	0.17	0.05	0.33	0.05	0.091
24:0[3]	0.19	0.06	0.26	0.04	0.227
∑16:0[3]	219.2	11.09	234.6	12.89	0.308
∑BCFA[3]	8.08	0.41	8.19	0.66	0.563
∑SFA[3]	446.8	18.49	489.3	16.49	0.190

[1] Control diet.

[2] Supplemented diet.

[3] Variables for which a lognormal distribution was used. Raw mean (± se) values are presented.

BCFA: branched chain saturated fatty acids, SFA: saturated fatty acids.

∑BCFA = 12:0*iso*, 12:0*anteiso*, 13:0*iso*, 14:0*iso*, 14:*anteiso*, 15:0*iso*, 16:0*iso*, 16:0*anteiso*; ∑SFA = 4:0, 6:0, 8:0, 10:0, 11:0, 12:0, 13:0, 14:0, 15:0, 16:0, 17:0, 18:0, 20:0, 21:0, 22:0, 23:0 and 24:0.

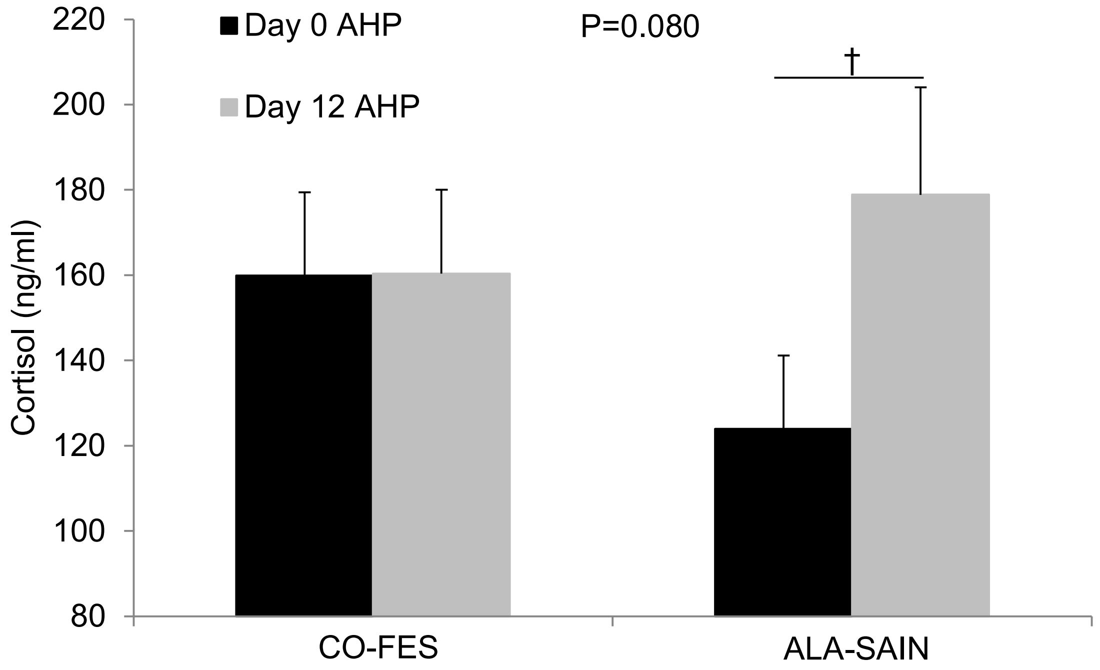

Figure S1: Differences between plasma cortisol concentration of lambs at the beginning and the end of the AHP according to pre-natal diet.

Supplementary Material S1 Independent variables included in the statistical model analysing each dependent variable

Statistical models for ewe haematology, and plasma cortisol, IL-2, leptin and lysozyme concentrations right after birth, and colostrum physicochemical measurements, chemical composition, fatty acid composition, leptin, lysozyme, and IgG concentrations included ewe age (years), pre-partum body condition, milk production during last lactation (l), gestation duration (d), birth type (single/twin) and pre-natal diet (CO-FES/ALA-SAIN) as fixed effects, and pre-partum pen as a random effect. Colostrum total solids concentration and SCC models additionally included the effect of time (0h vs. 24h post-partum) and its interaction with pre-natal diet.

Statistical models for lamb BW at birth included gestation duration, birth type, pre-natal diet, lamb sex, and the interaction between pre-natal diet and lamb sex as fixed effects, and pre-partum pen as a random effect. Lamb BW until 35d of age, and of lamb BW during AHP were analysed using repeated measures analysis, including lamb birth BW (g) as covariate, gestation duration, birth type, pre-natal diet, lamb sex (male/female), time, and 2-way interactions as fixed effects, and post-partum pen as random effect.

Lamb serum IgG concentration and AEA were analysed using repeated measures analysis, with BW and colostrum IgG concentration at birth as covariates, gestation duration, birth type, pre-natal diet, lamb sex, time, and 2-way interactions as fixed effects, and post-partum pen as random effect.

Plasmatic cortisol during AHP was analysed using repeated measures analysis, with sex, pre-natal diet, time, and 2-way interactions as fixed effects and post-partum pen as random effect.

Statistical models for behaviour variables included lamb sex, pre-natal diet and their interaction as fixed effects, and test group and post-partum pen as random effects.

Statistical model for plasma cortisol at the end of behaviour tests included pre-test concentration as covariate, lamb sex, pre-natal diet, and their interaction as fixed effects; and post-partum pen as random effect.

Skinfold thickness and temperature, and plasma cortisol, IL-2, IL-10, and IL-1β concentrations during IC were analysed using repeated measures analysis, with pre-IC values as covariates, lamb sex, pre-natal diet, time, and 2-way interactions as fixed effects, and post-partum pen as random effect.
